# Genetic Interaction of *SEEDSTICK, GORDITA* and *AUXIN RESPONSE FACTOR* 2 during Seed Development

**DOI:** 10.3390/genes12081189

**Published:** 2021-07-30

**Authors:** Dario Paolo, Gregorio Orozco-Arroyo, Lisa Rotasperti, Simona Masiero, Lucia Colombo, Stefan de Folter, Barbara A. Ambrose, Elisabetta Caporali, Ignacio Ezquer, Chiara Mizzotti

**Affiliations:** 1 Dipartimento di BioScienze, Università degli Studi di Milano, 20133 Milano, Italy; dario.paolo@ibba.cnr.it (D.P.); gregorio.orozco@unimi.it (G.O.-A.); lisa.rotasperti@unimi.it (L.R.); simona.masiero@unimi.it (S.M.); lucia.colombo@unimi.it (L.C.); elisabetta.caporali@unimi.it (E.C.); juan.ezquer@unimi.it (I.E.); 2 Laboratorio Nacional de Genómica para la Biodiversidad, Centro de Investigación y de Estudios Avanzados del Instituto Politécnico Nacional (CINVESTAV-IPN), Irapuato CP 36824, Guanajuato, Mexico; stefan.defolter@cinvestav.mx; 3 The New York Botanical Garden, Bronx, NY 10458, USA; bambrose@nybg.org

**Keywords:** ARF, MADS, maternal tissues, seed development, transcription factors

## Abstract

Seed development is under the control of complex and coordinated molecular networks required for the formation of its different components. The seed coat development largely determines final seed size and shape, in addition to playing a crucial role in protecting the embryo and promoting germination. In this study, we investigated the role of three transcription factors known to be active during seed development in *Arabidopsis thaliana*: SEEDSTICK (STK) and GORDITA (GOA), two MADS-domain proteins, and AUXIN RESPONSE FACTOR 2 (ARF2), belonging to the ARF family. Through a reverse genetic approach, we characterized the seed phenotypes of all the single, double and triple loss-of-function mutants in relation to seed size/shape and the effects on metabolic pathways occurring in the seed coat. This approach revealed that dynamic networks involving these TFs are active throughout ovule and seed development, affecting the formation of the seed coat. Notably, while the genetic interaction among these genes results in synergies that control the promotion of cell expansion in the seed coat upon pollination and production of proanthocyanidins, functional antagonists arise in the control of cell proliferation and release of mucilage.

## 1. Introduction

In spermatophytes, seeds are the vehicle on which plants rely for their propagation and reproductive success. In this context, seed size represents one of the most important parameters that influence plant fitness. For instance, given a certain amount of energy, small-seeded species produce more seeds than large-seeded ones, resulting in higher colonization abilities. Nevertheless, seedlings derived from large-seeded species better tolerate environmental stresses [1,2]. In agriculture, food grains have been subjected to artificial selection and breeding for number, size, among other qualities, with the result that modern crops usually have more, larger and heavier seeds compared to wild-type relatives [3,4,5,6].

Seeds are composed of three different structures: the embryo and the endosperm, formed upon double fertilization, and the seed coat that differentiates from ovule integuments.

The seed coat defines the perimeter of the inner seed cavity and it serves as a primary barrier versus the external conditions, protecting the next generation from biotic and abiotic stresses [7]. The seed coat of *Arabidopsis thaliana* is composed of five layers: two layers derive from the ovule’s outer integuments and three from the inner ones. The outermost integuments (oi) consist of the abaxial (outer, oi2) and adaxial (inner, oi1) epidermis, while inner teguments (ii) contains three layers (from external to internal: ii2, ii1′ and ii1) [8]. The innermost layer of inner integuments (ii1) is called endothelium [9].

The development of the seed coat and formation of the seed cavity is based on both cell division and expansion [10] and, in Arabidopsis, the final seed size is reached around 6 days after pollination (DAP) [11]. Interestingly, differences in specialized metabolism are found among seed coat layers: the endothelium, ii1, accumulates different flavonoids, in particular proanthocyanidins (PAs), while the outer epidermis, oi2, accumulates mucilage.

Given the importance of the seed coat, we characterized the role of three transcription factors (TFs) known to be active during seed coat development and crucial to control seed size and seed coat differentiation: SEEDSTICK (STK), GORDITA (GOA) and AUXIN RESPONSE FACTOR 2 (ARF2). The MADS-domain TF STK is involved in ovule identity determination [12,13,14], transmitting tract development [15,16], and fruit development [17]; the role of STK in seed development has also been dissected, showing its pivotal function in seed abscission [14,18] and in the control of PAs biosynthesis in the endothelium [19]; STK is also a determinant for the mechanical properties of the cell wall in seeds [20]. It is known that loss-of-function *stk* mutants have smaller seeds with respect to wild type [14], but no defects in plant fertility. In seeds, STK promotes cell cycle via *E2Fa*, a mechanism that has been proposed as crucial to achieve proper seed size [21]. Finally, together with the B-sister gene *ARABIDOPSIS B-SISTER* (*ABS*), STK is required for endothelium differentiation [13,22]. The only other B-sister gene in Arabidopsis is *GOA*, which is involved in cell expansion during fruit and seed development, as inferred by the characterization of the *goa* mutant, where both seeds and fruits are larger with respect to wild type [23,24]. In addition to increased size, *goa* seeds have a defective seed coat, as shown by increased permeability to tetrazolium salts compared to wild type, suggesting defects in seed dormancy, germination and protection [25]. Other defects are detectable in the oi1 layer of the seed coat: in *goa* mutant, the cells of this layer are longer and narrower than wild type ones [24]. Finally, we investigated ARF2, a B3-type transcription factor of the Auxin-Responsive-Factor (ARF) family [26]; ARF2 regulates integument growth as its loss-of-function mutation (*arf2*) determines a dramatic increase in seed size and seed mass [27]. The *arf2* phenotype is due to extra cell division in the integuments causing the formation of an enlarged seed coat [27,28]. ARF2 activity strongly depends on hormonal signaling and, thus, it has been suggested as a possible molecular link between the brassinosteroids and the auxin cascades in organ size determination [26,29].

In order to provide further insights into the transcriptional regulatory networks controlling seed development, we characterized in detail the seed phenotype of *stk*, *goa* and *arf2* mutants, as well as of their combinations, revealing a novel genetic interaction among these genes.

## 2. Materials and Methods

### 2.1. Plant Material and Growth Conditions

Arabidopsis wild type (ecotype Columbia, Col-0) and transgenic lines were grown on soil at 22 °C under short-day (8 h light/16 h dark) and long-day conditions (16 h light/8 h dark). *stk-2* contains a 74 nt insertion close to the splice site of the 3rd intron [14]; *goa-1* contains a T-DNA insertion in the 4th intron [24]; *arf2-8* (SALK_108995) was previously identified as a mutant allele of *ARF2* [27].

### 2.2. Genotyping

PCR-based genotyping of wild type and mutant alleles was performed with the following primer pairs: *STK* and *stk-2* Atp_204/Atp_561; *GOA* Atp_3987/Atp_3988; *goa-1* Atp_3987/Atp_2060; *ARF2* Atp_3666/Atp_3667; *arf2-8* Atp_3666/Atp_1212 (Supplemental Table S1).

### 2.3. Seed Size Measurement

Three weeks after manual pollinations, mature dry seeds were photographed with a Leica stereo microscope equipped with Leica DFC280 camera and imaging software LAS AF 2.2.0 (Leica UK Ltd., Milton Keynes, UK). Image J software was used to calculate seed size. At least 250 seeds were measured for each genotype.

Detailed seed coat analysis was performed staining the seeds following the protocols previously reported [30,31]. Seeds were imaged with a Nikon A1 laser scanning confocal microscope. Image J software was used to calculate the length of the cells. At least 6 seeds were measured for each genotype.

### 2.4. Tissue Staining and Microscopy

The vanillin assay for PAs detection was performed as described previously [32]. Vanillin (vanilaldehyde- V1104, Sigma-Aldrich, St. Louis, MO, USA) condenses with PAs and flavan-3-ol precursors to give a bright-red product in acidic conditions. Microscopic observations were performed using a Zeiss Axiophot D1 microscope equipped with differential interface contrast (DIC) optics. Images were recorded with an Axiocam MRc5 camera (Zeiss) using the Axiovision program (version 4.1).

Ruthenium-red (0.01% *w*/*v* Sigma-Aldrich) staining and evaluation of mucilage extrusion on mature seeds was performed upon 60 min of water imbibition as previously described [33]. Seeds were photographed with a Leica stereo microscope equipped with Leica DFC280 camera and imaging software LAS AF 2.2.0 (Leica UK Ltd., Milton Keynes, UK).

Scanning electron microscopy of mature dry seeds was performed as previously described [34] by gold coating them using a sputter coater (SEMPREP2; Nanotech) followed by observation with a LEO 1430 scanning electron microscope (LEO Electron Microscopy).

## 3. Results and Discussion

### 3.1. ARF2, GOA and STK Control Seed Size and Shape

**Figure 1:**
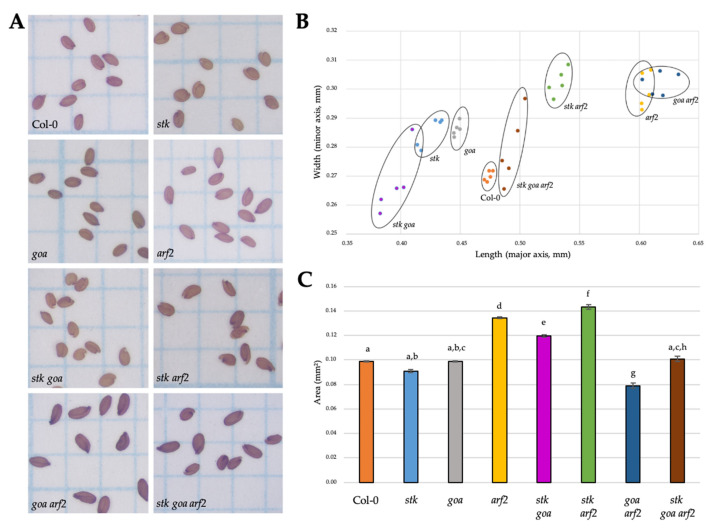
Size comparison of mature seeds. (**A**) Comparison of mature seeds three weeks after manual pollination of wild type Col-0, *stk*, *goa*, *arf2*, *stk goa*, *stk* *arf2*, *goa arf2* and *stk goa arf2* plants with pollen of the same genotype. (**B**) Data distribution of mature seeds based on length (major axis) and width (minor axis). Each dot represents a biological replicate with a mean of at least 50 seeds coming from a single parent. Five biological replicates were produced from populations coming from independent parental lines and similar values were obtained. (**C**) Seed area of different mutants combination. Values represent the average area (mm^2^) of 10 biological replicates with at least 100 seed measurements for each genotype. Error bars represent s.e. of the sample. Letters above the bars display statistical differences based on Tukey HSD test at *p* ≤ 0.05.

It was previously reported that STK, ARF2 and GOA affect seed size: *stk* mutant seeds are smaller than wild type ones, while *arf2* and *goa* seeds are bigger [14,24,27]. To study a possible genetic interaction, we characterized the mature seed phenotypes of the single mutants *stk*, *goa* and *arf2*, as well of the double mutant *stk goa*, *stk arf2*, *goa arf2* and the triple mutant *stk goa arf2* (Figure 1A).

In order to quantify the differences in seed size, mature dry seeds were harvested three weeks after manual pollination and analyzed. Manual pollination was necessary because self-pollination is impaired in the *arf2* mutant due to longer pistils in the flowers [27], a phenotype that is persistent in all the analyzed mutant combinations that include *arf2*. Since *arf2* mutants (and all the mutant combinations that included *arf2*) show poor self-fertility, the only way to effectively compare seed size and yield is by performing manual pollination (also referred to as restricted pollination). Using this strategy, Hughes et al. (2008) [35], did not detect significant changes on the number of produced seeds per silique, although they reported a significant increase in the mean seed weight for the *arf2* mutant with respect to wild type. Therefore, for our experiments, we characterized all *arf2* mutant seeds from manual pollinations.

We described the phenotype of all mutants by measuring the average length (major axis) and width (minor axis) and the seed area of mature seeds (Figure 1B,C). All the single, double and triple mutants differ from wild type for seed length with *stk*, *goa* and the double mutant *stk goa* seeds shorter than the wild type, while all the other mutant combinations are longer respect to the control. *arf2* single mutant and *goa arf2* produce the longest seeds among all the other combinations, while the length of *stk arf2* seeds is reduced, although not as much as in the wild-type control. Regarding the seed’s width, all the single and the double mutants are wider than the wild type, with the exception of *stk goa*. These data suggest that *stk* and *goa* single mutants have a rounder shape in comparison to the ovoid seeds of wild type. Finally, more phenotypic variability characterizes the seeds of the triple mutant *stk goa arf2*, as shown in Figure 1. 

Differently from what previously reported [24], we observed that in comparison to wild type, *goa* seeds have a reduced length and increased width, resulting in a length/width ratio of 1.56, which determines a rounder shape with respect to the wild type (length/width ratio 1.75) (Supplemental Figure S1).

Our data indicate that *STK* and *GOA* promote seed length while *ARF2* represses it. Moreover, seeds analyses from the double and the triple mutant combinations indicate that ARF2 is epistatic to GOA and STK in the control of seed length. While the reduced *stk goa* seed length suggests that *STK* functions synergistically with *GOA* to control seed length. Regarding the seed width, all these transcription factors act as repressors.

We also analyzed the overall impact of these mutations on seed area (Figure 1C). In terms of seed area in the single mutants, we found no statistical differences from *stk* and *goa* with respect to WT, while *arf2* presented statistically bigger seed area with respect to WT. In double mutant combinations we found that *stk goa* and *stk arf2* had the biggest seed area while the *arf2 goa* combination had very short seeds. Interestingly, any impact on seed area was ameliorated in the triple *stk arf2 goa* as the triple mutant was similar to WT. 

### 3.2. STK, GOA and ARF2 Control Cell Proliferation and Expansion in the Seed Coat

It is well established that in Arabidopsis final seed size largely depends on the formation of the seed cavity [36,37]. We previously showed that STK is expressed in developing ovules and seeds [13,38]: in particular the analysis of the *pSTK::STK-GFP* line revealed that STK protein is present in the seed coat until 4 DAP [19]. GOA was previously shown to be expressed in the outer integument and embryo sac in mature ovules and its expression is maintained in the outer integument and chalazal region in the developing seeds [24]. ARF2 expression was previously reported in embryos [27] and in developing seeds [28]. We evaluated the activity of a putative ARF2 promoter during seed development with reporter construct *pARF2::3XGFP-SV40* and we observed the GFP signal, driven by the ARF2 promoter, throughout the seed coat after fertilization (Supplemental Figure S2).

With the aim to investigate the genetics of the observed size-phenotypes, we performed crosses with wild type pollen. This was done to exclude seed-size related phenotypes that could be ascribed, at least partially, to the size of embryo and endosperm. To better analyze the morphology of the seed coat, all the crosses were stained and analyzed with confocal laser scanning microscopy. This technique was performed in order to quantify differences between the mutants in terms of cell proliferation (average cell number counted) and cell expansion (average cell length). We took in consideration two layers of the seed coat: the outermost one (epidermis, oi2) and the innermost one (endothelium, ii1).

**Figure 2:**
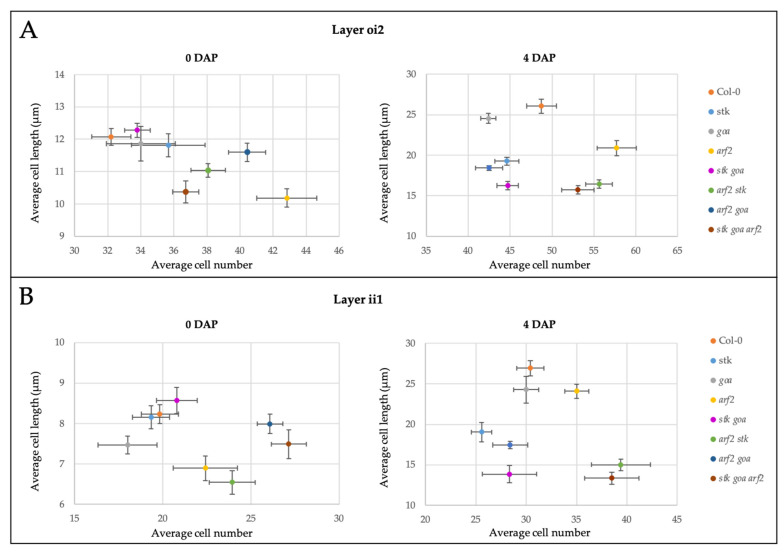
Maternal control of seed coat development. Size distribution of cell number (major axis) and cell length (minor axis) in oi2 (**A**) and ii1 (**B**) layers in ovules (0 DAP) and in seeds derived from crosses with WT pollen (4 DAP). Each dot represents a mean of at least 6 seeds. Error bars represent s.e.

Measurements of seed coat cells were performed at 0 DAP and at 4 DAP (Figure 2); the latter is a time point where the increase in dimension of the seeds reaches a peak, and the final volume of the inner seed cavity is almost established [36]. All differences reported and discussed in this section were found to be statistically significant (*p* ≤ 0.05, Supplemental Table S2). At 0 DAP we detected no differences with respect to the wild type in *stk*, *goa* and *stk goa* mutants, neither in terms of cell length nor of cell number. On the contrary, *arf2* and all the combinations lacking *ARF2* have ovules with shorter cells, but in higher number with respect to the wild type.

At 4 DAP we observed that in *stk* × WT seeds, the seed coat has impaired elongation. In both layers examined the length of *stk* × WT cells is shorter than the wild type ones, suggesting that *stk* seed phenotype is mainly due to a reduction in cell expansion rather than in cell division, and that defects are of maternal origin. On the other hand, *goa* × WT seeds do not differ from control. As we reported at 0 DAP, also at 4 DAP the seed coat of *arf2* × WT seeds has a higher number of shorter cells.

The seed coat of *stk goa* × WT has significant defects in elongation, as for both the layers examined the average cell length is even more reduced than the single mutant *stk* × WT, hinting that STK and GOA act synergistically in the promotion of cell elongation in the developing seed coat. This functional synergism is further enhanced by *ARF2*, as shown by the more drastic phenotypes observed whenever *ARF2* is lacking, both in comparison to the wild type and to *arf2* × WT. On the other hand, when looking at cell number, the over-proliferation phenotype observed in *arf2* × WT is also found in *stk arf2* × WT and in *stk goa arf2* × WT but not in *arf2 goa* × WT.

These measurements suggest a complex network controlling cell proliferation and expansion in the seed coat both before and after pollination. In particular, the central role of ARF2 as a repressor of cell proliferation [27] is confirmed by our analysis. Based on the characterization of the double and triple mutants, ARF2 action on cell proliferation was found to be partially epistatic to STK, but not to GOA. We recently demonstrated that STK controls cell cycle progression in the developing seed coat, via the positive regulation of *E2Fa* [21]. While the reduction of cell number in comparison to wild type observed in *stk* × WT crosses was not found to be significant, this could trace back to a size effect bias, due to the difficulties of screening a significantly large number of crosses with such a methodology (Cohen’s d = 0.78 (oi2) and 1.3 (ii1)).

In addition to their antagonistic role on the regulation of cell proliferation, STK, GOA and ARF2 synergistically promote cell expansion. We had previously shown how STK determines the biochemical and structural properties of cell walls in the seed coat and the increased stiffness of *stk* mutant seed coat would explain the defects in cell elongation [20], a phenotype we confirmed being maternally inherited. Other studies have linked GOA to cell expansion, as shown by the enlarged fruits and seeds of the *goa* mutant [24]. However, the maternal control of seed size exerted by GOA was never tested and, based on our analysis, cell expansion was not observed in *goa* × WT seed coat cells, ruling out the hypothesis that *GOA* might only act maternally to determine seed size.

### 3.3. Functional Role of STK, GOA and ARF in the Control of PAs Accumulation and Mucilage Release in the Seed Coat

The production and accumulation of PAs (also known as condensed tannins) is an example of how the specialized metabolism of the seed coat is involved in the protection of the embryo. PAs are polymers of flavan-3-ols with functions in protection from UV-radiation, deterrence from herbivores and ascribed to microbial activities [39]. In Arabidopsis seeds, PAs are synthesized and accumulated in the innermost seed coat layer, the endothelium [32], while in the external layer of the seed coat the synthesis of the mucilage occurs. STK controls seed coat development by the coordination of the biosynthesis and release of the mucilage [20] and the synthesis and accumulation of PAs [19]. To verify whether the above-described genetic network that controls seed size and shape is also controlling these specialized metabolisms, we also explored the role of GOA and ARF2 in these processes.

**Figure 3:**
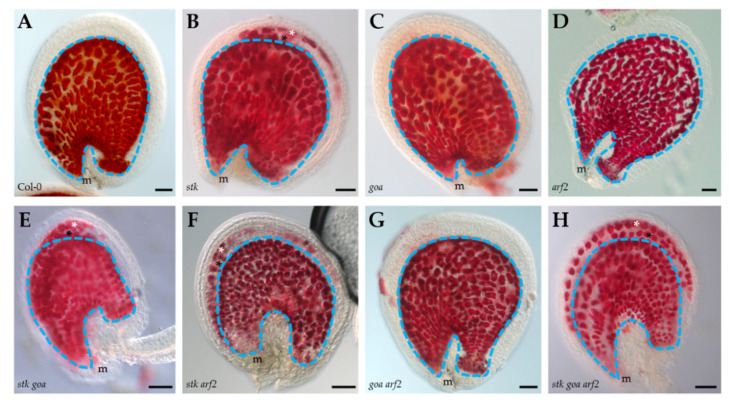
PAs accumulation in wild type and mutant seeds. Pink staining indicates PAs accumulation. Accumulation of PAs in the endothelium is marked by blue outline, while ectopic accumulation is marked by black asterisks (second layer) and white asterisks (third layer). For each one of the analyzed genotypes, *n* > 25 seeds were examined and representative image of the phenotype is presented; WT ((**A**), *n* = 58), *stk* ((**B**), *n* = 62), *goa* ((**C**), *n* = 76), *arf2* ((**D**), *n* = 26), *stk goa* ((**E**), *n* = 33), *stk arf2* ((**F**), *n* = 43), *goa arf2* ((**G**), *n* = 38), *stk goa arf2* ((**H**), *n* = 25). Scale bar = 50 μm. m, mycropile.

In order to elucidate if PAs accumulation was affected in *arf2* and *goa* mutant seeds, we performed a PAs staining in seeds with vanillin [40]. In wild type seeds, PAs accumulation starts in the micropylar region of the endothelium at 1 DAP and progresses gradually to the chalazal region, until it is visible in all endothelial cells from 5 to 6 DAP (Figure 3A). As previously described [19], *stk* seeds progressively accumulate PAs in the endothelium with similar temporal and spatial patterns to wild type, ectopic accumulation of PAs also occurs, specifically in the third layer of the seed coat and, to a minor extent in the second layer (Figure 3B). On the contrary, single mutant seeds of *goa* and *arf2* and the double mutant *goa arf2* do not have alteration in PAs accumulation (Figure 3C,D,G). Interestingly, the ectopic PAs accumulation is even more drastic in *goa stk* and *arf2 stk* double mutants than in the *stk* single mutant. Coherently *stk goa arf2* triple mutant strongly accumulates PAs in the second and third layer of the seed coat (Figure 3E,F,H). Overall, these data indicate that ectopic PAs accumulation is an additive phenotype and indicate that the molecular mechanism confining PAs accumulation to the endothelium depends on the direct transcriptional repression exerted by STK on the anthocyanin reductase gene *BANYULS/BAN* in the other layers of the seed coat [19]. The characterization of double and triple mutants points at a functional synergism that also includes GOA and ARF2 in the fine regulation of PAs metabolism, representing a novel finding on the role of these TFs in the control of specialized metabolism of the seed.

Another crucial specialized pathway that occurs in the seed coat leads to the production and secretion of mucilage, a sticky substance composed of polar glycoproteins that serves important ecological processes such as water stress tolerance, allelopathy, or facilitation of germination [41].

We have demonstrated that *stk* displays defects in mucilage release upon water imbibition [20] and to unveil the possible redundant role of the TFs examined here in mucilage release, we performed a pectin staining with Ruthenium red on mature hydrated seeds [42].

**Figure 4:**
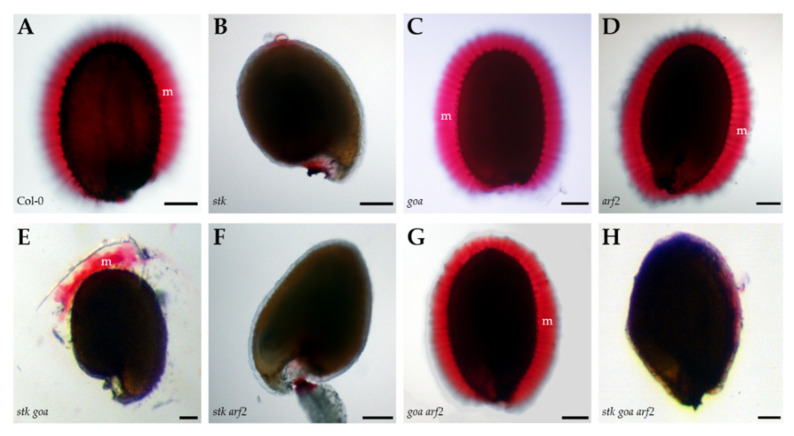
Mucilage extrusion assay. Wild type (**A**) and mutant mature seeds (*goa* (**C**), *arf2* (**D**) and *goa arf2* (**G**)) show mucilage-specific staining upon hydration and ruthenium-red treatment. Positive stain for pectin indicates the adherent mucilage layer. The cellulose network of the extruded mucilage and the more densely stained “rays” radiating from each columella can be seen. No capsule of mucilage is apparent on *stk* (**B**), *stk arf2* (**F**) and *stk goa arf2* (**H**) seeds stained after imbibition in water. Partial extrusion of adherent mucilage was observed on *stk goa* (**E**) seeds. For each one of the analyzed genotypes, *n* > 25 seeds were examined and a representative image of the phenotype is presented; WT (*n* = 28), *stk* (*n* = 25), *goa* (*n* = 29), *arf2* (*n* = 30), *stk goa* (*n* = 20), *stk arf2* (*n* = 26), *goa arf2* (*n* = 28), *stk goa* arf2 (*n* = 27). Scale bar=100 μm. m, mucilage.

Wild type, *goa*, *arf2* and *arf2 goa* seeds similarly release mucilage after one hour of water imbibition (Figure 4A,C,D,G). On the contrary, no mucilage release is visible in the *stk* mutant seeds. The structural defects of the cell walls in *stk* seeds [20] could explain this phenotype, as improper mucilage release could be linked to increased mechanical resistance of the outer primary cell wall [33]. Moreover, impaired release of mucilage was observed for all the mutant combinations lacking STK function (Figure 4B,F,H), with the partial exception of *stk goa* (Figure 4E), where we observed a limited and patchy mucilage release. This observation could be linked to the alterations of seed coat permeability described for *goa* seeds, that might partially overcome the impaired release of mucilage. These data confirm that STK function is critical for the correct release of mucilage, whilst ARF2 and GOA plays a minor role in this process. 

### 3.4. Morphology of the Seed Coat Epidermis

**Figure 5:**
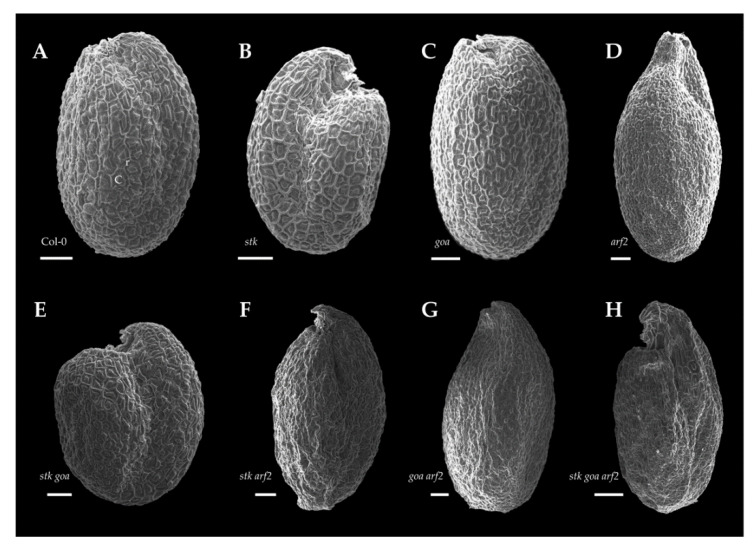
Scanning electron microscopy of seeds. SEM imaging of mature seeds. (**A**) Wild type Col-0 epidermal seed coat cells have hexagonal shape with a clear radial cell wall and columella. The seed coat epidermal cell morphology of *stk* (**B**), *goa* (**C**) and *stk goa* (**E**) is very similar to wild type ones. On the contrary, *arf2* (**D**), *stk arf2* (**F**), *goa arf2* (**G**) and *stk goa arf2* (**H**) present defects on the radial cell wall formation and the columella appears more elongated than in wild type. For each one of the analyzed genotypes, *n* > 25 seeds were examined, a representative image of the phenotype is presented. Scale bar = 100 μm. c, columella; r, radial cell wall.

The morphology of the seed coat epidermis was studied by performing scanning electron microscopy (SEM), examining the surface of dry seeds. Morphologically, for single mutants *stk* and *goa*, as well as for the double *stk goa*, epidermal cells were indistinguishable from wild type (Figure 5A–C,E), as they are similarly characterized by an hexagonal shape with the columella at the center and thickened radial cell walls.

On the contrary, the seed coat epidermal cells of *arf2* have several structural differences when compared with wild type: *arf2* epidermal cells show an irregular shape and are difficult to discriminate because of irregularities of the radial cell walls (Figure 5D). In some cells, the radial cell wall is completely absent and only the apparently normal columella is discernible. In the *stk arf2* and *goa arf2* double mutants and *stk goa arf2* triple mutant seed coat, defects in the form of the cells and the radial cell walls are slightly more severe than in *arf2* (Figure 5F–H), with obvious alteration of overall seed shape.

This morphological characterization indicates that ARF2 is a major determinant of epidermal differentiation in seeds, a role that was previously unreported for this TF, a process that does not involve STK and GOA.

## 4. Conclusions

**Figure 6:**
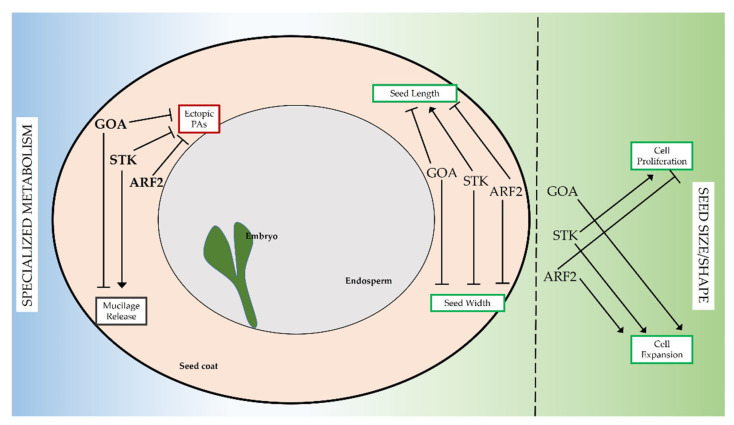
Network involving STK, GOA and ARF2. Throughout seed development STK, GOA and ARF2 create dynamic networks to control the production of specialized metabolites (PAs and mucilage, left) or define seed size and shape (right). An arrow indicates an activation, a bar at the end of an edge indicates an inhibition.

For spermatophytes, fitness strongly relies on production of viable seeds, whose development traces back to complex molecular regulatory networks. We have shown how the three transcription factors STK, GOA and ARF2, known to be involved in reproductive development, take part in dynamic networks during seed coat development such as control of seed size/shape and the specialized metabolic pathways of PAs production and mucilage release (Figure 6).

## Data Availability

The data presented in this study are available on request from the corresponding author.

## Supplementary Materials

The following are available online at www.mdpi.com/article/10.3390/genes12081189/s1, Figure S1: Seed length/width ratio. The ratio between seed length and width is indicated. Values represent the average of 5 biological replicates with at least 50 seeds for each genotype. Error bars represent s.e. of the sample. Letters above the bars display statistical difference based on Tukey HSD test at *p* ≤ 0.05. Figure S2: Expression pattern of *pARF2::SV40-3xGFP*, *pSTK::STK-GFP* and *pGOA:GUS* in the seed coat. Table S1: Primer pairs used in this work. Table S2: Data related to size distribution reported in Figure 5. In the central diagonal the average means of cell number and cell length in oi2 and ii1 layers in ovules (0 DAP) and in seeds derived from crosses with WT pollen (4 DAP) is reported. Each mean represents at least 10 seeds. The statistical differences among the different genotype was calculated based on Tukey HSD test (* = *p* ≤ 0.05; ** = *p* ≤ 0.01).
