# Supplementary material for: Genetic Interaction of SEEDSTICK, GORDITA and AUXIN RESPONSE FACTOR 2 during Seed Development
Source: Genes (Basel). 2021 Jul 30;12(8):1189. doi: 10.3390/genes12081189 (PMC8393894; doi:10.3390/genes12081189)
Supplement: Supplementary file 1 [file genes-12-01189-s001.zip › Supplemental Material (Paolo et al 2021)/supplementary Figures.pdf]

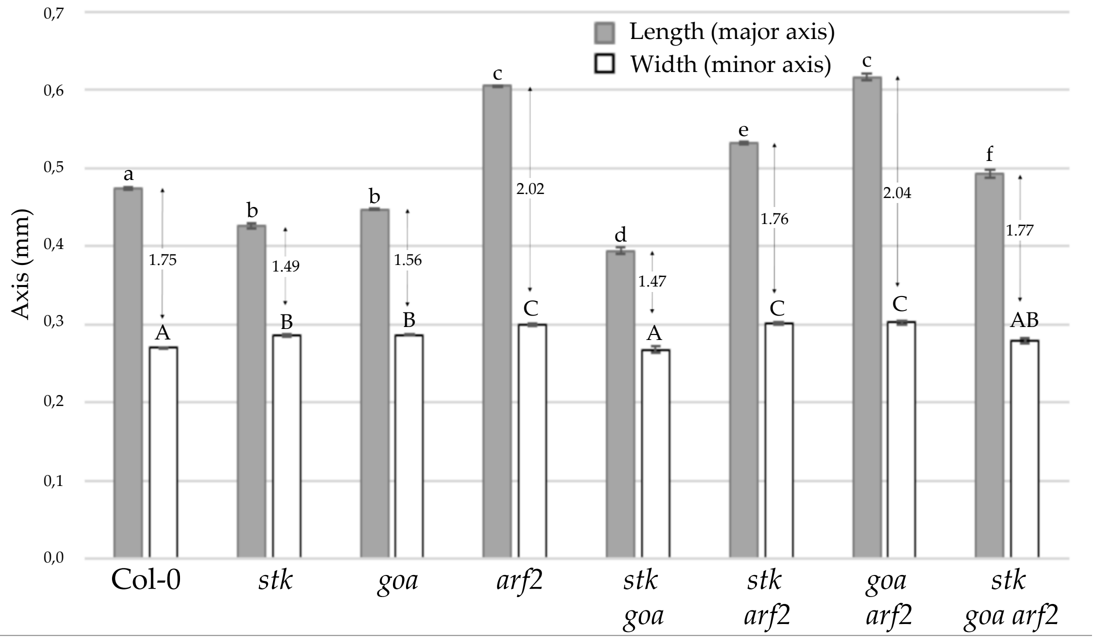

Figure S1: Seed length/width ratio. The ratio between seed length and width is indicated. Values represent the average of 5 biological replicates with at least 50 seeds for each genotype. Error bars represent s.e. of the sample. Letters above the bars display statistical difference based on Tukey HSD test at $p \leq 0.05$.

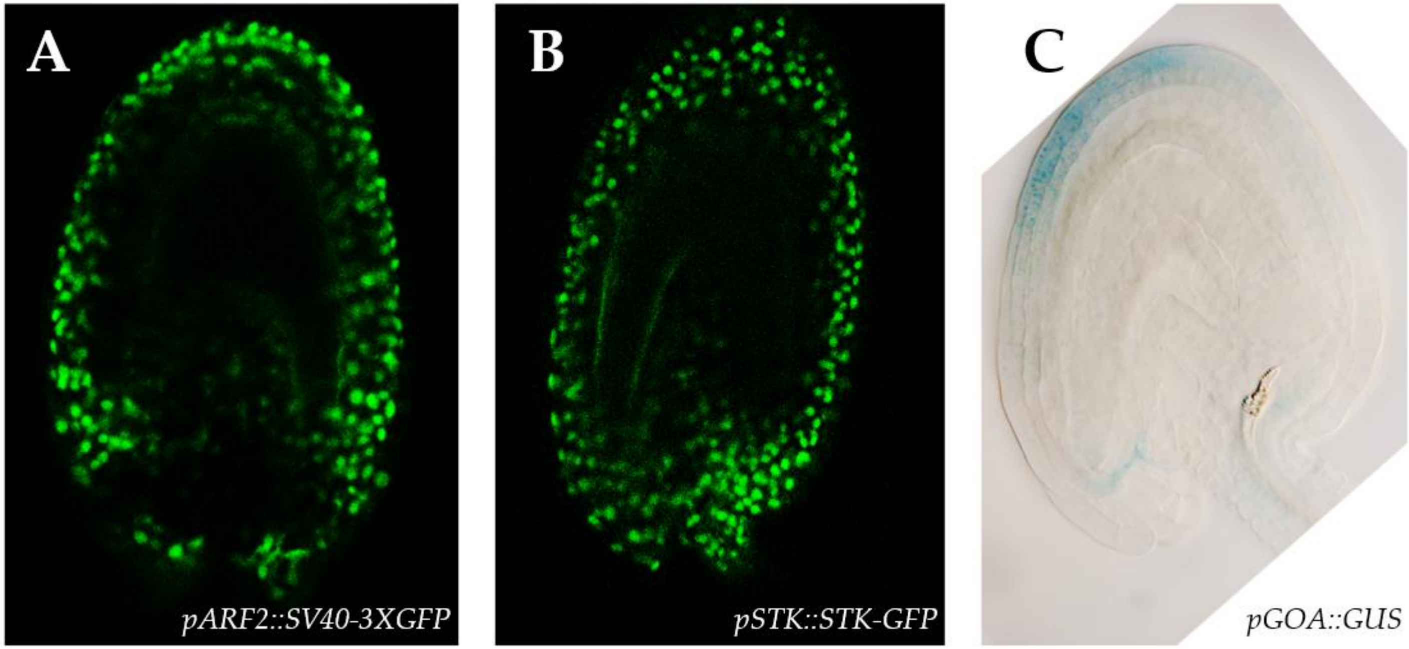

Figure S2: Expression pattern of *pARF2::SV40-3xGFP*, *pSTK::STK-GFP* and *pGOA:GUS* in the seed coat. Table S1: Primer pairs used in this work. Table S2: Data related to size distribution reported in Figure 5. In the central diagonal the average means of cell number and cell length in oi2 and ii1 layers in ovules (0 DAP) and in seeds derived from crosses with WT pollen (4 DAP) is reported. Each mean represents at least 10 seeds. The statistical differences among the different genotype was calculated based on Tukey HSD test (* = $p \leq 0.05$; ** = $p \leq 0.01$).
