# Supplementary material for: Genetic Interaction of SEEDSTICK, GORDITA and AUXIN RESPONSE FACTOR 2 during Seed Development
Source: Genes (Basel). 2021 Jul 30;12(8):1189. doi: 10.3390/genes12081189 (PMC8393894; doi:10.3390/genes12081189)
Supplement: Supplementary file 1 [file genes-12-01189-s001.zip › Supplemental Material (Paolo et al 2021)/Table S1.pdf]

Table S1. Primer pairs used in this work.

Genotype	Primers	Sense primers (5′ → 3′)	Antisense primers (5′ → 3′)
STK* and *stk-2	*Atp_204 / Atp_561*	GCTTGTTCTGATAGCACCAACACTAGCA	GGAACTCAAAGAGTCTCCCATCAG
GOA	*Atp_3987/Atp_3988*	ACATGCTCGAGCTCGCAAG	ATTCGCCGGCGCAGGATC
goa-1	*Atp_3987/Atp_2060*	ACATGCTCGAGCTCGCAAG	ACTAGTATGTTACGTCCTGTAGAAAC
ARF2	*Atp_3666 / Atp_3667*	TGAGTCCTGTTCCAATGCC	GAAAGATCCACTGAACGGC
arf2-8	*Atp_3666 / Atp_1212*	TGAGTCCTGTTCCAATGCC	GCGTGGACCGCTTGCTGCAACT
