# Supplementary material for: Genetic Interaction of SEEDSTICK, GORDITA and AUXIN RESPONSE FACTOR 2 during Seed Development
Source: Genes (Basel). 2021 Jul 30;12(8):1189. doi: 10.3390/genes12081189 (PMC8393894; doi:10.3390/genes12081189)
Supplement: Supplementary file 1 [file genes-12-01189-s001.zip › Supplemental Material (Paolo et al 2021)/Table S2.pdf]

Table S2. Data related to size distribution reported in Figure 5. In the central diagonal the average mean of cell number and cell length in oi2 and ii1 layers in ovules (0 DAP) and in seeds derived from crosses with WT pollen (4 DAP) is reported. Each mean represents at least 10 seeds. The statistical difference among the different genotype was calculated based on Tukey HSD test (* = $p \leq 0.05$; ** = $p \leq 0.01$).

0 DAP	oi2	Number		Col-0	*stk*	*goa*	*arf2*	*stk goa*	*stk arf2*	*goa arf2*	*stk goa arf2*
			Col-0	32.21 ± 5.18	n.s.	n.s.	**	n.s.	**	**	n.s.
			stk	n.s.	35.67 ± 5.43	n.s.	*	n.s.	n.s.	n.s.	n.s.
			goa	n.s.	n.s.	34 ± 4.64	*	n.s.	n.s.	n.s.	n.s.
			arf2	**	*	*	42.83 ± 6.29	**	n.s.	n.s.	n.s.
			stk goa	n.s.	n.s.	n.s.	**	33.79 ± 2.89	n.s.	*	n.s.
			stk arf2	**	n.s.	n.s.	n.s.	n.s.	38.07 ± 4.04	n.s.	n.s.
			goa arf2	**	n.s.	n.s.	n.s.	*	n.s.	40.43 ± 4,24	n.s.
			stk goa arf2	n.s.	n.s.	n.s.	n.s.	n.s.	n.s.	n.s.	36.71 ± 2.06

0 DAP	oi2	Length		Col-0	*stk*	*goa*	*arf2*	*stk goa*	*stk arf2*	*goa arf2*	*stk goa arf2*
			Col-0	12.08 ± 1.13	n.s.	n.s.	**	n.s.	n.s.	n.s.	**
			stk	n.s.	11.82 ± 0.88	n.s.	*	n.s.	n.s.	n.s.	n.s.
			goa	n.s.	n.s.	11.86 ± 1.19	*	n.s.	n.s.	n.s.	n.s.
			arf2	**	*	*	10.18 ± 0.98	**	n.s.	n.s.	**
			stk goa	n.s.	n.s.	n.s.	**	12.28 ± 0.81	*	**	n.s.
			stk arf2	n.s.	n.s.	n.s.	n.s.	*	11.04 ± 0.82	n.s.	n.s.
			goa arf2	n.s.	n.s.	n.s.	n.s.	**	n.s.	11.6 ± 1.06	n.s.
			stk goa arf2	**	n.s.	n.s.	**	n.s.	n.s.	n.s.	10.37 ± 0.91

0 DAP	ii1	Number		Col-0	*stk*	*goa*	*arf2*	*stk goa*	*stk arf2*	*goa arf2*	*stk goa arf2*
			Col-0	19.83 ± 4.42	n.s.	n.s.	n.s.	n.s.	n.s.	**	**
			stk	n.s.	19.33 ± 3.12	n.s.	n.s.	n.s.	n.s.	*	*
			goa	n.s.	n.s.	18 ± 4.72	n.s.	n.s.	n.s.	**	*
			arf2	n.s.	n.s.	n.s.	22.42 ± 6.3	n.s.	n.s.	n.s.	n.s.
			stk goa	n.s.	n.s.	n.s.	n.s.	20.79 ± 4.32	n.s.	*	n.s.

			stk arf2	n.s.	n.s.	n.s.	n.s.	n.s.	23.94 ± 5.2	n.s.	n.s.
			goa arf2	**	*	**	n.s.	*	n.s.	26.07 ± 2.73	n.s.
			stk goa arf2	**	*	*	n.s.	n.s.	n.s.	n.s.	27.14 ± 2.61

0 DAP	ii1	Length		Col-0	*stk*	*goa*	*arf2*	*stk goa*	*stk arf2*	*goa arf2*	*stk goa arf2*
			Col-0	8.23 ± 0.97	n.s.	n.s.	*	n.s.	**	n.s.	n.s.
			stk	n.s.	8.16 ± 0.85	n.s.	n.s.	n.s.	**	n.s.	n.s.
			goa	n.s.	n.s.	7.47 ± 0.64	n.s.	n.s.	n.s.	n.s.	n.s.
			arf2	*	n.s.	n.s.	6.90 ± 1.05	**	n.s.	n.s.	n.s.
			stk goa	n.s.	n.s.	n.s.	**	8.57 ± 1.22	**	n.s.	n.s.
			stk arf2	**	**	n.s.	n.s.	**	6.55 ± 1.16	**	n.s.
			goa arf2	n.s.	n.s.	n.s.	n.s.	n.s.	**	7.99 ± 0.91	n.s.
			stk goa arf2	n.s.	n.s.	n.s.	n.s.	n.s.	n.s.	n.s.	7.49 ± 0.93

4 DAP	oi2	Number		Col-0	*stk*	*goa*	*arf2*	*stk goa*	*stk arf2*	*goa arf2*	*stk goa arf2*
			Col-0	48.73 ± 5.92	n.s.	n.s.	**	n.s.	*	n.s.	n.s.
			stk	n.s.	44.6 ± 4.55	n.s.	**	n.s.	**	n.s.	**
			goa	n.s.	n.s.	42.4 ± 2.8	**	n.s.	**	n.s.	**
			arf2	**	**	**	57.71 ± 6.13	**	n.s.	**	n.s.
			stk goa	n.s.	n.s.	n.s.	**	44.71 ± 3.40	**	n.s.	*
			stk arf2	*	**	**	n.s.	**	55.6 ± 5.1	**	n.s.
			goa arf2	n.s.	n.s.	n.s.	**	n.s.	**	42.5 ± 3.99	**
			stk goa arf2	n.s.	**	**	n.s.	*	n.s.	**	53.1 ± 6.1

4 DAP	oi2	Length		Col-0	*stk*	*goa*	*arf2*	*stk goa*	*stk arf2*	*goa arf2*	*stk goa arf2*
			Col-0	26.08 ± 2.89	**	n.s	**	**	**	**	**
			stk	**	19.28 ± 1.54	**	n.s	*	*	n.s	**
			goa	n.s	**	24.56 ± 1.91	**	**	**	**	**
			arf2	**	n.s	**	20.89 ± 2.52	**	**	n.s	**
			stk goa	**	*	**	**	16.24 ± 1.41	n.s	n.s	n.s
			stk arf2	**	*	**	**	n.s	16.43 ± 1.66	n.s	n.s

				Col-0	*stk*	*goa*	*arf2*	*stk goa*	*stk arf2*	*goa arf2*	*stk goa arf2*
			goa arf2	**	n.s	**	n.s	n.s	n.s	18.42 ± 0.72	n.s
			stk goa arf2	**	**	**	**	n.s	n.s	n.s	15.72 ± 1.65

4 DAP	ii1	Number		Col-0	*stk*	*goa*	*arf2*	*stk goa*	*stk arf2*	*goa arf2*	*stk goa arf2*
			Col-0	30.4 ± 4.33	n.s	n.s	n.s	n.s	*	n.s	n.s
			stk	n.s	25.56 ± 3.05	n.s	n.s	n.s	**	n.s	*
			goa	n.s	n.s	30 ± 3.55	n.s	n.s	*	n.s	n.s
			arf2	n.s	n.s	n.s	35 ± 3.16	n.s	n.s	n.s	n.s
			stk goa	n.s	n.s	n.s	n.s	28.33 ± 6.59	*	n.s	*
			stk arf2	*	**	*	n.s	*	39.4 ±.9.26	*	n.s
			goa arf2	n.s	n.s	n.s	n.s	n.s	*	28.4 ± 3.85	n.s
			stk goa arf2	n.s	*	n.s	n.s	*	n.s	n.s	38.5 ± 8.58

4 DAP	ii1	Length		Col-0	*stk*	*goa*	*arf2*	*stk goa*	*stk arf2*	*goa arf2*	*stk goa arf2*
			Col-0	26.94 ± 3	**	n.s	**	**	**	**	**
			stk	**	19.04 ± 3.64	*	*	*	n.s	n.s	*
			goa	n.s	*	24.28 ± 4.61	n.s	**	**	**	**
			arf2	**	*	n.s	24.08 ± 2.34	**	**	**	**
			stk goa	**	*	**	**	13.84 ± 2.64	n.s	n.s	n.s
			stk arf2	**	n.s	**	**	n.s	15 ± 2.26	n.s	n.s
			goa arf2	**	n.s	**	**	n.s	n.s	17.43 ± 1.05	n.s
			stk goa arf2	**	*	**	**	n.s	n.s	n.s	13.33 ± 2.31
